# Differentiated Evaluation of Extract-Specific Evidence on *Cimicifuga racemosa*'s Efficacy and Safety for Climacteric Complaints

**DOI:** 10.1155/2013/860602

**Published:** 2013-08-25

**Authors:** A.-M. Beer, A. Neff

**Affiliations:** ^1^Department of Naturopathy, Blankenstein Hospital, Im Vogelsang 5-11, 45527 Hattingen, Germany; ^2^Department of Gynecology, Lübbecke Hospital, Virchowstraße 65, 32312 Lübbecke, Germany

## Abstract

Past reviews on *Cimicifuga racemosa* (CR) without differentiation between extracts, quality, and indication altogether led to inconsistent data. Therefore, for the first time, we meet the requirements of the system's logic of evidence-based phytotherapy by taking into consideration extracts, pharmaceutical quality (reflected in a regulatory status as medicinal product), and indication. A literature search for clinical studies examining CR's efficacy and safety for menopausal complaints was conducted. The results were sorted by type of extract, regulatory status, and indication. Accordingly, Oxford Levels of Evidence (LOE) and Grades of Recommendation (GR) were determined. CR extracts demonstrated a good to very good safety in general, on estrogen-sensitive organs and the liver. However, only registered CR medicinal products were able to prove their efficacy. Best evidence was provided by the isopropanolic CR extract (iCR): the multitude of studies including more than 11,000 patients demonstrated consistent confirmatory evidence of LOE 1b (LOE 1a for safety) leading to GR A. The studies on the ethanolic extract BNO 1055 including more than 500 patients showed exploratory evidence of LOE 2b resulting in GR B. A positive benefit-risk profile is stated and limited to *Cimicifuga racemosa* products holding a marketing authorisation for treating climacteric complaints.

## 1. Introduction

Frequently climacteric complaints, especially vasomotor symptoms (hot flashes, sweatings) and vaginal dryness, are treated with hormone therapy (HT) [1, 2]. Comprehensive clinical studies in the 1990s (Women's Health Initiative Study (WHI) and Women's International Study of long Duration Oestrogen after Menopause (WISDOM)) have demonstrated, however, that women who were treated with hormone therapy for climacteric complaints for several years had a significantly increased risk of breast cancer, cardiovascular events, pulmonary embolism, and dementia compared to placebo [3–5]. Both patients as well as physicians have been looking for effective and safe alternatives [3, 6]. Different extracts from *Cimicifuga racemosa* (Black Cohosh, Traubensilberkerze) are clinically used for climacteric complaints [7]. The monograph for *Cimicifuga racemosa* by the Herbal Medicinal Product Committee (HMPC) of the European Medicines Agency (EMA) stipulates a clear therapeutic benefit for three specific extracts and confirms a positive benefit-risk profile [8]. The extract production with a proven pharmaceutical quality under GMP conditions is the prerequisite.

The rhizome of *Cimicifuga racemosa* with attached roots is harvested in fall after the fruit has ripened and is used fresh or in dried form. The rhizome of *Cimicifuga racemosa* contains cycloartenol-type triterpene glycosides (amongst others actein, cimicifugoside, cimiracemoside A, and 23-epi-26-deoxyactein) and phenylpropanoids such as hydroxycinnamic acids. Further ingredients include alkaloids, N-methylserotonin, starch, fatty acids, resin, and tannins [9]. The isoflavone formononetin, once described as an ingredient, could not be detected uniformly in newer studies of extracts of *Cimicifuga racemosa* [10, 11]. 

The evidence regarding the aggregate of the different types of *Cimicifuga racemosa* extracts available on the market has been evaluated rather differently by various reviews. In this context, the heterogeneity of the data [12], contradicting results [13] or the inconsistency of data [14] have been criticised. Problems arise from the fact that previous reviews included all types of *Cimicifuga* preparations, whether approved as medicinal products or other products (food supplements or individualized single preparations) and their use for most different indications, without any differentiation. 

However, none of these reviews took into consideration the logical system of phytotherapy, which requires a differentiation by type of extract, pharmaceutical quality, and indication. Herbal extracts are multiple-substance mixtures with varying composition, depending on the extractant used and further distinctions specific for manufacture, harvest, or cultivation. This can lead to different efficacies [15].

In contrast to food supplements or dietary foods, state-of-the-art proof of quality, efficacy, and safety is mandatory for the registration of herbal medicinal products. Strict quality criteria are applied especially for herbal medicinal products in all areas starting with drug harvesting followed by production of extract and finished product to the point of stability testing [16]. Quality and quality control are essential factors, which guarantee a stable efficacy and safety. The standardization of the manufacturing processes and a multitude of quality control steps from seed to finished product are prerequisites for the reproducibility of efficacy and safety demonstrated in studies and warrant a consistently high and certified quality of the medicinal product to both therapist and user.

Consequently, a differentiated review, evaluated by type of extract, pharmaceutical quality, and indication, which meets the characteristics and corresponds to the logical system of phytotherapy, is imperative. This review, therefore, differentiates clearly between registered and standardized medicinal products, on the one hand, and not registered/unspecified products, on the other. By means of a systematic review and assessment of the available evidence [17], it differentiates and evaluates the efficacy and the safety of phytopharmaceuticals from *Cimicifuga racemosa* for the treatment of climacteric complaints.

## 2. Methodology

### 2.1. Search Algorithm

We searched the databases MEDLINE, BIOSIS, EMBASE, EMBASE Alert, and PubMed for clinical studies with *Cimicifuga racemosa*. Furthermore, our research was complemented by a manual search in the library of authors. Goal of the literature search was the identification of clinical studies exploring the efficacy (search 1: efficacy) and safety of *Cimicifuga racemosa*. In reference to safety, 3 searches were conducted in order to identify both studies with results regarding the general safety (frequency and severity of adverse effects) as well as studies investigating the safety in regard to estrogen-sensitive organs (breast, uterus) and the liver.

The search was executed via a free text search using keywords linked with AND/OR/NOT-operators as well as in combination with the corresponding keywords (e.g., Medical Subject Headings) of the corresponding database. Used keywords were *Cimicifuga racemosa*/Traubensilberkerze/black cohosh/*Actaea racemosa*, clinical trial/clinical study/klinische Studie, Review/meta analysis/Meta Analyse, efficacy/Wirksamkeit, side effect/Nebenwirkung/adverse reaction/adverse drug/adverse event/adverse effect/ADR/UAW/interaction/Interaktion/Wechselwirkung/safety/Sicherheit/toxicity/Toxizität/intoxication/Intoxikation/poison, breast/Brust/mamma/uterin/uterus/Gebärmutter/tumor/tumour/cancer/hormon/estrogen/Östrogen, Leber/liver/hepat, case report/Fallstudie.

In order to ensure the currentness of the evidence, the range of the publications was limited to the years 2000–2012.

### 2.2. Selection of Publications

The search results were preselected based on their titles and abstracts. Criteria for inclusion and exclusion were predetermined (Table 1). References, which were considered potentially relevant, were then viewed as full text. The result of the first selection included all references, which suggested meeting the inclusion and exclusion criteria. A final assessment occurred after the full texts had been submitted to the secondary selection. If full texts were available, furthermore a supplemental manual search was performed within the bibliography of the publications.

The data obtained from the secondary selection of the included references were assessed and evaluated in summary based on their methodology and the quality of their studies.

### 2.3. Inclusion, Exclusion, and Stratification Criteria

For the assessment of the efficacy, this review only lists clinical studies in which women with neurovegetative and/or psychic climacteric complaints were treated with the investigated phyto-pharmaceutical for a minimum of 3 months. For a complete assessment of the evidence, the criterion “clinical study” included all types of studies (randomized/controlled, meta-analyses of randomized/controlled studies, open/controlled, open/uncontrolled, case-control studies, and epidemiological cohort studies). In reference to the safety assessment, the duration of treatment was not limited. In the controlled studies, the comparative interventions included placebo, hormone preparations/tibolone, fluoxetine, and different dosages of the study preparation as well as the comparison of a *Cimicifuga* mono-preparation versus a combination of the same extract with another active ingredient. For the assessment of the efficacy of interventions against climacteric complaints, placebo-controlled studies are essential. Therapy regimes with hormones/tibolone were also included as comparative intervention, since these are standard therapies for climacteric complaints. In recent years, effects of selective serotonin-reuptake inhibitors against vasomotor and psychic climacteric complaints have been described; therefore, this comparative intervention has also been included.

We did not conduct any explicit search on the effect (preclinical studies, mode of action). Secondary literature such as reviews, general surveys, comments, discussions, and conference presentations as well as the description of individual case reports/casuistics have been excluded. Clinical studies whose primary endpoint was outside the registered indication for *Cimicifuga racemosa* (such as bone metabolism, anxiety disorder, cognition, and tamoxifen-induced complaints) were not included in reference to the efficacy. In regard to the question whether *Cimicifuga racemosa* is a safe treatment option for breast cancer patients with climacteric complaints, studies conducted with these patients were included for assessment. Search results yielding studies with combined preparations containing more than two active ingredients were not included, since a definite attribution of efficacy and safety is not possible for multiple-substance-combinations.

Patient-relevant endpoints were the reduction of neurovegetative and/or psychic climacteric symptoms, the frequency and severity of adverse events, and the influence on breast/uterus/breast-cancer risk or laboratory values (such as gonadotropins, ovarian hormones, and liver function parameters). A change of the symptomatology was measured based on different scales such as the Kupperman Menopause Index (KMI), the Menopause Rating Scale (MRS), the Hamilton Depression Scale (HAMD), and frequency of symptoms or diverse scales on health-related quality of life. The selection was not limited to certain scales.

Only publications in English or German have been included in this systematic analysis.

A significant point of this review is the differentiation of results both by indication as well as by type of extract and the regulatory status. The results were sorted and reported accordingly.

## 3. Results

### 3.1. Literature Search on the Efficacy

**Figure 1:**
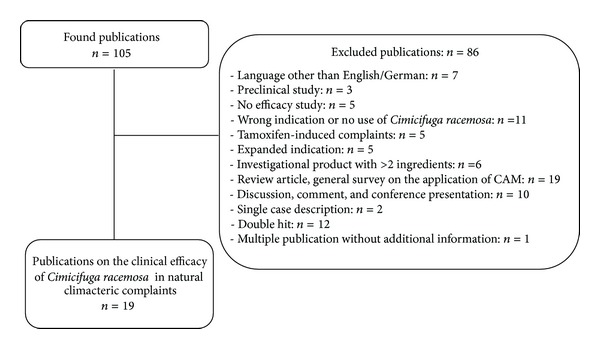
Results of the literature search on efficacy.

A total number of 105 references were identified. After reviewing abstracts and full publications, 86 search results were excluded in accordance with the inclusion and exclusion criteria (Figure 1).

19 full publications on a total of 18 clinical studies, which investigated the efficacy of *Cimicifuga racemosa* for natural climacteric complaints, remain.

### 3.2. Literature Search on Safety

For the assessment of the safety of *Cimicifuga racemosa*, 3 individual literature searches (general safety, safety on estrogen-sensitive organs, and liver safety) were conducted. If one of those individual searches yielded references whose content matched one of the other individual searches, these were allocated accordingly.

#### 3.2.1. Literature Search on General Safety

**Figure 2:**
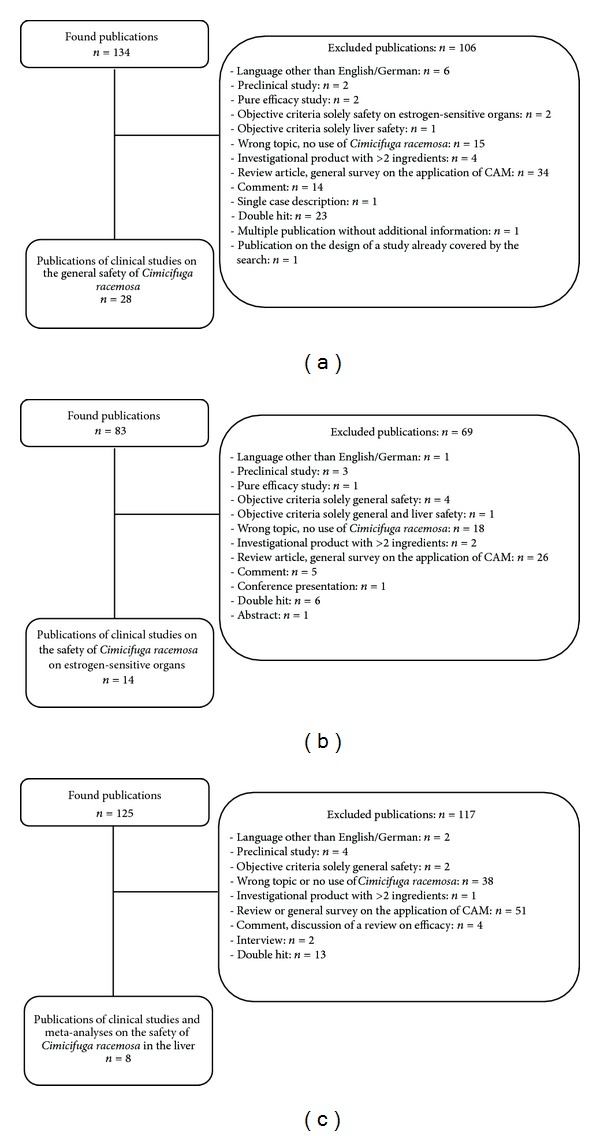
(a) Results of the literature search on general safety. (b) Results of the literature search on safety on estrogen-sensitive tissue. (c) Results of the literature search on liver safety.

A total of 134 references were identified. According to the inclusion and exclusion criteria, 106 of these search results were excluded (Figure 2(a)). In the end, we had found 28 publications concerning the general safety of *Cimicifuga racemosa*. Furthermore, we found 1 additional publication, which however exclusively deals with general safety, in our search regarding the drug safety in estrogen-sensitive organs.

Additionally, 2 publications were related to questions of drug safety in the liver and another 7 publications to the safety on estrogen-sensitive organs. Six publications additionally involved aspects of safety both in the liver and estrogen-sensitive organs.

#### 3.2.2. Literature Search Concerning Safety on Estrogen-Sensitive Tissue

A total of 83 references were identified. As per the inclusion and exclusion criteria, 69 references were excluded (Figure 2(b)). Fourteen publications concerning the safety of *Cimicifuga racemosa* in reference to estrogen-sensitive organs remained.

In addition, 4 of these publications also gathered data on general safety and 3 on the liver safety as well as general safety.

#### 3.2.3. Literature Search Concerning Liver Safety

Of initially 125 references, 117 were excluded as per the inclusion and exclusion criteria (Figure 2(c)). Eight publications remained, of which 7 referred to clinical studies. Furthermore, we found one meta-analysis of randomized, controlled studies.

Two of the publications additionally contained data on the general safety. Four publications contained additional data both on the general safety and the safety on estrogen-sensitive organs.

### 3.3. Summary of the Results of the Literature Search

#### 3.3.1. Efficacy

In total, 19 publications from 18 clinical studies have been identified, in which 10,284 patients were treated with *Cimicifuga racemosa* against natural climacteric complaints. Of these, in 15 clinical studies 10,121 patients (98.5%) were treated with a registered medicinal product, while in 3 studies a total of 163 patients (1.5%) was treated with *Cimicifuga*-products that had not been registered as medicinal products.

Concerning the special isopropanolic *Cimicifuga racemosa* extract (iCR), 9 original publications resulting from 9 clinical studies were found [18–26]. Two of these publications investigated the combination of iCR and St. John's Wort in comparison to the monotherapy with iCR [25] or placebo, respectively [26]. Both preparations are medicinal products registered in several countries. In these 9 studies, a total of 9,391 patients were treated with iCR, 6,126 patients with the monotherapy and 3,265 patients with the combination preparation. Thus, more than 91% of all patients treated in clinical studies with *Cimicifuga racemosa* received the iCR-extract.

Furthermore, 1 publication of a clinical study conducted with a combination of *Cimicifuga racemosa* and St. John's Wort, which is distributed in Korea as medicinal product, has been found [27]. In this study, 47 patients were treated with the preparation. The publication did not contain any information on the extractant.

Three publications on a total of 2 clinical studies could be identified for the ethanolic extract BNO 1055 [28–30]. This is a medicinal product that has been registered in several countries. In both studies together, a total of 420 patients (4% of all patients treated in clinical studies) were treated with the preparation.

Furthermore, 3 individual studies on different ethanolic extracts, which all have been registered as medicinal products, have been found. These include the extract Ze 450 (registered in Switzerland) [31], a standard extract for generics (Cr 99) [32], and a Turkish preparation [33]. In these studies, 120, 83, or 60 patients, respectively, were treated with the corresponding preparation.

Furthermore, 3 individual studies conducted with US-American *Cimicifuga* preparations without marketing authorisation as medicinal products have been identified. These concerned one preparation whose extractant was not mentioned [34], one ethanolic extract [35], and one ethanolic extract, which was exclusively used as study medication [36]. The brand name for the unspecified extract can be found among US-American *Cimicifuga* food supplements. In the individual studies, 61, 80, or 22 patients, respectively, were treated with the individual *Cimicifuga* preparations.

#### 3.3.2. Safety

A total of 41 full publications have been identified, which are based on 35 clinical studies and one meta-analysis of 5 published randomized, controlled studies. The publications furthermore include 5 reanalyses of clinical studies with additional information. Twenty-five clinical studies as well as the meta-analysis concerned registered medicinal products, 10 studies concerned unregistered products or preparations with unknown regulatory status or unspecified preparations. Within the context of these clinical studies, safety-relevant aspects were studied in a total of 13,492 users of *Cimicifuga racemosa*. Among those, 11,961 patients (88.6%) received registered medicinal products, 1,531 patients (11.4%) used *Cimicifuga* preparations not registered as medicinal products or unspecified preparations.

Concerning the isopropanolic *Cimicifuga racemosa* extract (iCR), we retrieved a total of 19 publications [18–23, 25, 26, 37–47]. Two of these investigated the combination of iCR and St. John's Wort in comparison to monotherapy with iCR [25] or placebo [26], respectively. The 19 publications are based on 17 clinical studies and 1 meta-analysis of 5 randomized, controlled studies already identified through the search [37]. One publication covers the second analysis of mammogram findings from a clinical study and their comparison with the findings from a comparable patient cohort obtained within the context of a placebo-controlled study with hormone therapy and tibolone [38]. On the whole, 11,054 patients (81.9%) were treated in the clinical studies with iCR, 7,789 with the monopreparation and 3,265 with the combination.

For the Korean combination preparation from *Cimicifuga racemosa* and St. John's Wort, the publication we already identified for the aspect “efficacy” also delivers safety-relevant information [27].

Regarding the ethanolic extract BNO 1055, 6 publications have been identified, which are based on 4 clinical studies [28–30, 48–50]. In these studies, a total of 597 patients (4.4%) were treated with the preparation.

The 3 studies already identified in the efficacy search for other registered preparations with ethanolic extracts also covered safety aspects [31–33].

Ten clinical studies refer to (mostly US-American) products not registered as medicinal products. These include the 3 publications already identified in the efficacy search [34–36], which also described safety-relevant aspects. Regarding the study of Newton et al. [35], 2 second analyses have been published [51, 52]. Furthermore, 2 publications on ethanolic extracts have been identified [53, 54] as well as 5 publications on extracts whose extractant has not been specified [55–59].

In 2 publications found for the isopropanolic extract (iCR), some patients, in addition to those who were treated with iCR, were treated with extracts, which were not specified in detail [45, 46].

## 4. Discussion

### 4.1. Answering the Question of Efficacy

Based on current, GCP-compliant studies and a clear separation by extracts and indications, today's response to the question of efficacy is highly differentiated.

When differentiating by regulatory status, it becomes obvious that all registered medicinal products containing *Cimicifuga racemosa* investigated in clinical studies uniformly demonstrate as a minimum exploratory evidence for their efficacy. In particular the isopropanolic extract (iCR) as monotherapy and in combination with St. John's Wort as well as the Korean *Cimicifuga*-St. John's Wort combination actually yielded confirmatory evidence for their efficacy. In contrast, US-American extracts, not registered as medicinal products, in the majority (2 out of 3 studies) could not yield any evidence for efficacy (Table 2). Evidence for efficacy clearly and significantly depends on the regulatory status as medicinal product, when analyzing the totality of all studies as well as when only analyzing those studies designed for confirmatory evidence (Tables 3(a) and 3(b)).

As early as in the 1980s, several clinical studies investigated the efficacy and safety of the first *Cimicifuga* preparation introduced in Germany in 1956 (Remifemin) for the indication of climacteric neurovegetative complaints [60]. Although the studies were conducted in a time period prior to the introduction of good clinical practice- (GCP-) criteria and prior to the publication of the monograph for *Cimicifuga racemosa* by the German Commission E [61], the “old” study results are clearly confirmed by the results of the “new” GCP-compliant studies.

Only 2 extracts were investigated in several GCP-compliant clinical studies for their efficacy. These are the isopropanolic extract iCR (9 publications from 9 studies with a total of 9,391 patients) and the ethanolic extract BNO 1055 (3 publications from 2 studies with a total of 420 patients). For both extracts therefore not only Levels of Evidence but also Oxford Grades of Recommendation can be derived. For all other extracts only one individual publication each is available.

#### 4.1.1. Medicinal Products with the Isopropanolic Extract (iCR)

The efficacy of the iCR extract has been proven by 4 randomized, controlled studies [18, 20, 21, 26] and is further supported by 2 controlled as well as 3 uncontrolled studies with proof of efficacy as primary objective [19, 22–25].

A significantly superior improvement of climacteric complaints was demonstrated in a total of 304 patients after 3 months of treatment with the standard dose of iCR (40 mg) versus placebo. The superiority was especially significant in women in menopausal transition, and the strongest effect could be achieved for vasomotor complaints [18]. In comparison to low-dose transdermal hormone therapy in a total of 64 women, a comparably strong, significant improvement of vasomotor complaints, anxiety and depression could be achieved after 3 months [21]. Furthermore, in 3 months of therapy, a strong, significant improvement in the Kupperman Menopause Index, comparable to tibolone, was achieved in 244 Asian women. Since the iCR extract in this investigation, however, had the better safety profile, it was superior to tibolone in the benefit-risk profile [20]. After 3 months of treatment with the iCR extract, responder rates reached approximately 70–80% [19, 20].

In addition to vasomotor symptoms, many menopausal women also suffer from psychic complaints such as nervousness, irritability, anxiety, restlessness, or depressive moods [62, 63]. In these patients especially, the combination of St. John's Wort and *Cimicifuga* extract has been successfully used in clinical practice for decades. These clinical experiences have been confirmed by controlled clinical studies. In a randomized, controlled study in 301 women, the combination of iCR and St. John's Wort was used for 4 months. In comparison to placebo, both the Menopause Rating Scale as well as the Hamilton Rating Scale for Depression improved significantly [26]. In a controlled study with 6,141 patients who were treated for 6 months (or 736 patients who were treated up to 12 months, resp.), the combination of iCR and St. John's Wort proved to be significantly more effective than the iCR-monotherapy for psychic climacteric complaints [25].

Two larger, uncontrolled studies, in which 502 or 2,016 women, respectively, were treated for 3 months, demonstrated significant improvements of the Kupperman Menopause Index, which were most pronounced for vasomotor complaints [22, 23]. Another, uncontrolled study in 122 patients demonstrated a significant increase in the quality of life after 3 months of treatment [24].

**Figure 3:**
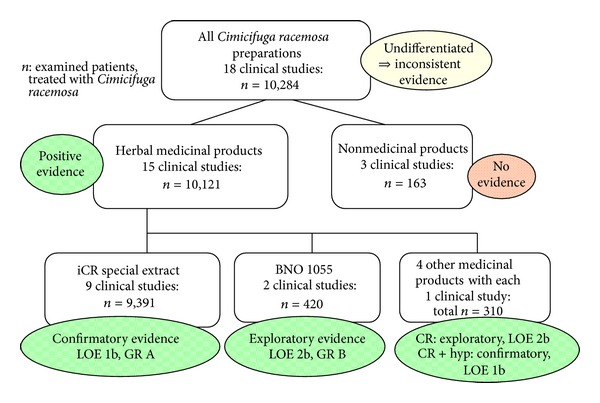
Efficacy data on *Cimicifuga* racemosa 2000–2012.

The randomized, controlled studies [18, 20, 21, 26] all qualify for an Oxford Level of Evidence 1b. Due to the consistent Level-1 data, a Grade of Recommendation A can be stated [17] (Figure 3).

#### 4.1.2. Medicinal Products with the Ethanolic Extract BNO 1055

Exploratory efficacy of this extract was demonstrated in a randomized, controlled study with a small case number, on which a reanalysis has been published [28, 29]. It has also been supported by a uncontrolled study [30].

In the controlled study with a total of 62 women, the improvement in the Menopause Rating Scale (MRS) for the ethanolic *Cimicifuga* extract was comparable to a hormone preparation versus placebo after 3 months. Due to the small case number (*n* = 20 in the *Cimicifuga* group), however, it just stayed below the level of significance. In the exploratory reanalysis, however, a significant superiority versus placebo in reference to daily sweats, waking at night, and the MRS-subscores *major climacteric complaints*, *somatic complaints,* and *mental score* could be demonstrated.

The uncontrolled study investigated the safety of the extract in the endometrium in 400 women for 12 months and also demonstrated a significant improvement of the Menopause Rating Scale II and the MRS II 4-week weighted score of hot flushes.

The controlled study of low quality yielded sufficient evidence for an Oxford Level of Evidence 2b. Due to the consistent Level-2 data, a Grade of Recommendation B may be postulated (Figure 3).

#### 4.1.3. Further Extracts with Marketing Authorization as Medicinal Product

For 4 further *Cimicifuga* medicinal products (three mono preparations and one combination with St. John's Wort) each with one publication of a randomized, controlled study, confirmatory (combination preparation) or at least exploratory evidence (mono preparations) for their efficacy was demonstrated.

After 3 months of therapy in a total of 180 women, the ethanolic extract Ze 450 was significantly superior versus placebo in the Kupperman Menopause Index. Furthermore, a higher dosage proved to be more effective in comparison to the standard dose [31]. The ethanolic extract Cr 99 was able to demonstrate its superiority versus placebo in the Kupperman Menopause Index and in the weekly weighted score of hot flashes after a 3-month therapy in a total of 125 women. The superiority was only significant, however, in a subgroup of women who suffered from stronger complaints (KMI > 20) at the beginning of the study [32]. An extract registered as a medicinal product in Turkey (ethanolic as per manufacturer's homepage) and the control substance fluoxetine demonstrated a significant improvement in the modified Kupperman Menopause Index, Beck's Depression Scale, and the RAND-36-QoL after 6 months in 120 patients. Concerning the improvement of the Kupperman Menopause Index and hot flashes as well as night sweats, the *Cimicifuga*-extract was significantly superior to fluoxetine. In the other scores, fluoxetine proved to be better. The quality of these studies allows to postulate a Level of Evidence 2b (Figure 3).

A Korean combination preparation from *Cimicifuga racemosa* and St. John's Wort yielded confirmatory evidence for its efficacy after 3 months of use: in comparison to placebo there was a significantly superior improvement in the Kupperman Menopause Index. A total of 89 women participated in this study. This study justifies a Level of Evidence 1b (Figure 3).

#### 4.1.4. Other Extracts without Marketing Authorization as Medicinal Product

For 3 US-American *Cimicifuga* preparations without marketing authorization as medicinal product, a total of 2 randomized, controlled and 1 open, uncontrolled study could be identified. The dosage information in the publications does not always clearly indicate whether it refers to the amount of extract or drug.

A 5-arm randomized, controlled study was conducted for 12 months with an ethanolic extract in a total of 351 women. Hereby, no significant differences could be found between the *Cimicifuga* preparation (daily dosage 160 mg) and placebo [35] in reference to the vasomotor symptoms. A 4-arm randomized, controlled study in a total of 89 women demonstrated no significant improvement of the frequency of vasomotor complaints in comparison to placebo after 12 months of treatment for another ethanolic extract (daily dose 128 mg) [36]. Merely an open, uncontrolled study in 61 women could demonstrate a significant improvement of the Kupperman Menopause Index after 3 months of treatment in comparison to baseline [34].

The controlled studies yielded no evidence for the efficacy of the used *Cimicifuga*-preparations, which prior to their use had not been subject to any control by regulatory drug approval procedures.

### 4.2. Answering the Question of Safety

#### 4.2.1. General Safety

Of the 41 publications identified in the literature search, 31 investigated aspects relevant for general safety (Table 4). Uniformly and independently from their regulatory status, they demonstrated that extracts from *Cimicifuga racemosa* have a good to very good tolerability. Overall, no side effects were recorded, which go beyond the known side effects listed in the HMPC monograph [8]. The placebo-controlled studies demonstrated that in regard to frequency and severity of adverse effects, *Cimicifuga racemosa* does not significantly differ from treatment with placebo [18, 26, 28, 32, 35, 36]. As also described in the HMPC-monograph, there are no generally known drug interactions for *Cimicifuga racemosa* [8]. This has been confirmed by 2 studies which did not find any clinically relevant interaction with cytochrome P-isoenzymes or P-glycoprotein [55, 56].

The safety data for the *Cimicifuga racemosa* preparations investigated in clinical studies can be assessed uniformly and in general confirm the safety of the used preparations.

#### 4.2.2. Safety in regard to Estrogen-Sensitive Tissue

Twenty two of the 41 publications investigated safety-relevant aspects under the therapy with *Cimicifuga racemosa* in reference to estrogen-sensitive tissues such as breast or uterus (Table 4). Under treatment with *Cimicifuga racemosa*, no clinically relevant changes of hormonal parameters (e.g., estradiol, FSH, and LH) were detected (e.g., [19, 21, 27, 36, 40, 52]). The endometrium is neither influenced by *Cimicifuga racemosa* [20, 21, 28, 30, 36, 43]. Mammographic breast density, breast cell proliferation, or cell morphology in the nipple aspirate fluid neither changed under *Cimicifuga* treatment [30, 34, 36, 38, 43]. Some smaller studies in breast cancer patients also demonstrated good tolerability of *Cimicifuga racemosa* in these patients [39, 41, 44, 49]. Several large case-control studies investigated the influence of *Cimicifuga racemosa* on breast cancer risk. A publication found no association between the application of *Cimicifuga racemosa* and breast cancer risk [59]. A US-American study demonstrated that breast cancer risk was reduced in users of *Cimicifuga racemosa* [46]. A study supported by the German Cancer Aid (Deutsche Krebshilfe e.V.) with more than 9,900 participating women showed no increase in breast cancer risk by ethanolic *Cimicifuga racemosa* extracts. For the isopropanolic *Cimicifuga racemosa* extract, this investigation even demonstrated a reduction of breast cancer risk [45]. This result was independent of lifestyle factors, tumor histology and tumor receptor status, and a longer duration of use improved risk reduction. A pharmacoepidemiological cohort study was able to demonstrate a slight reduction in the risk of recurrence for the isopropanolic *Cimicifuga racemosa* extract (*n* = 1,102 compared to *n* = 17,759 not treated with iCR) [47].

The entirety of the data clearly contradicts the existence of any estrogenic effects of *Cimicifuga racemosa*, which had been suggested decades ago. Under therapy, no adverse effects are to be expected on “critical” estrogen-sensitive organs such as breast or uterus. Based on the study results therefore, climacteric complaints in patients with breast cancer in their medical history can be treated with extracts of *Cimicifuga racemosa*. The most recent HMPC-monograph [8] confirms this assumption, provided that the cancer treating physician knows about the *Cimicifuga* therapy.

#### 4.2.3. Liver Safety

In 11 of 41 publications, liver function tests were performed and described prior to as well as during or at the end of the treatment with *Cimicifuga racemosa* extracts (Table 4). On the whole, no clinically relevant changes of liver function tests became evident (e.g., [18, 20, 21, 32, 48, 50, 58]). Also within the context of all the studies, no liver damage was clinically observed as adverse event. Significant evidence is available for the isopropanolic extract in the form of a meta-analysis of 5 randomized, controlled studies, in which liver safety parameters were recorded [37]. None of these revealed significant differences nor indications of a negative influence onto liver functions. An increase in the dosage compared to the HMPC recommendation or an increased duration of use, respectively, also demonstrated no significant effects.

In contrast to the findings from clinical studies, there are individual case reports, which suspect *Cimicifuga racemosa* within the context of hepatotoxicity. These include reports on food supplements, which are not subject to the same strict quality controls as medicinal products. For food supplements, for instance, impurities and adulterations (such as using cheaper Asian *Cimicifuga* species) are known [64]. Therefore, the reported suspected cases of liver side effects in connection with food supplements cannot be applied to registered *Cimicifuga* medicinal products in general.

A first analysis of internationally published and reported cases was conducted by the European Medicines Agency in 2006. It did not yield any certain causality. In 4 of 42 cases known worldwide, the causality was assessed as possible or probable, respectively [65]. A reanalysis of these 4 questionable cases by means of a liver-specific, validated algorithm was able to exclude a causal connection, however [66]. After 2006, further case reports have become known, which have been evaluated by different groups of experts. Depending on the used algorithm, one receives different causality assessments. When using the liver-unspecific Naranjo scale, a causal connection seems possible [67]. If, however, the assessment is based on the use of a liver-specific, validated algorithm, there is hardly any to no evidence at all for a hepatotoxic potential of *Cimicifuga racemosa* [68–72]. Experts in the field of hepatology point out the shortcomings of the Naranjo-scale as being not liver-specific and not validated for hepatotoxicity. They demand to apply only liver-specific causality assessment methods which are validated for liver toxicity (e.g., the CIOMS scale) in suspected cases of herb-induced liver injury [73].

#### 4.2.4. Extract-Specific Differences in the Safety Data

From extract to extract, differences have to be taken into consideration in regard to the number of included patients and objectives of the conducted studies.

Of all patients, who were monitored for side effects in clinical studies investigating any *Cimicifuga racemosa* preparation (*n* = 13,492), the majority were treated with registered medicinal products (*n* = 11,961). In 1,531 patients no registered medicinal products were used or the preparations were not specified sufficiently, in order to be able to conclude their regulatory status. Among the medicinal products, there were only 2 preparations, which were used in a variety of studies, the isopropanolic *Cimicifuga racemosa* extract (iCR) and the ethanolic extract BNO 1055.

**Figure 4:**
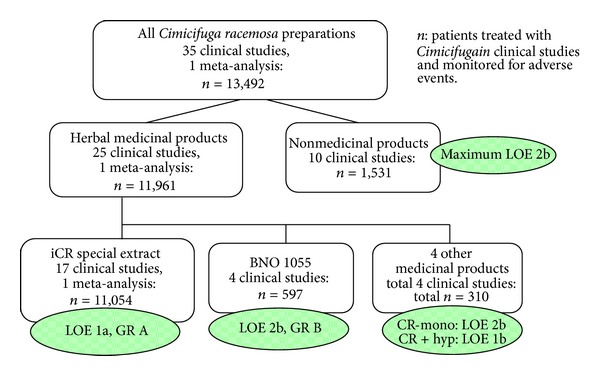
Safety data on *Cimicifuga racemosa* 2000–2012.

By far, most patients treated in clinical studies with a medicinal product (*n* = 11,054) received the isopropanolic *Cimicifuga racemosa* extract (iCR) among which 736 were observed for adverse events for a period of 12 months. Thus, both as monotherapy as well as in combination with St. John's Wort, the iCR extract is the only *Cimicifuga* extract which fulfills the requirements of the EU guideline ICH-E1 (CPMP/ICH/375/95) for the permission of a long-term therapy. For this extract, the state of the evidence is the best: among the 17 publications there are not only several high-quality, randomized, and controlled studies, but also a homogenous meta-analysis of 5 randomized, controlled studies. The safety data for the iCR-extract therefore corresponds to an Oxford Level of Evidence 1a, and based on the consistent study results a Grade of Recommendation A can be stated (Figure 4).

The ethanolic extract BNO 1055 was administered in altogether 597 patients. Among the publications, there is 1 randomized, controlled study (with low case number and quality, of which 2 exploratory reanalyses have been published), 1 controlled study and two uncontrolled studies. The status of the safety data allows an Oxford Level of Evidence 2b and Grade of Recommendation B (Figure 4).

The status of the data of other medicinal products in regard to drug safety corresponds to Oxford Level of Evidence 2b, for the Korean *Cimicifuga*-St. John's Wort combination to Level of Evidence 1b (Figure 4).

The evidence for the preparations not registered as medicinal products varies, the Level of Evidence regarding safety has to be considered maximally 2b (Figure 4).

### 4.3. Benefit-Risk Profile of *Cimicifuga racemosa*-Extracts—Differences between Registered Medicinal Products and Extracts without Marketing Authorization as Medicinal Product

While all *Cimicifuga racemosa* extracts used in clinical studies demonstrated a good to very good safety profile independently of their regulatory status, this is not the case in regard to efficacy. Here, only officially assessed, registered medicinal products yielded the proof of efficacy. All *Cimicifuga* medicinal products could at least prove exploratory efficacy for the registered indication of neurovegetative and psychic climacteric complaints, and the isopropanolic extract (iCR) and a Korean medicinal product additionally yielded confirmatory evidence. Consistent investigations have been conducted for the isopropanolic iCR-extract and the ethanolic BNO 1055 extract, so that for these extracts Grades of Recommendation can be determined (iCR: LOE 1b, GR A; BNO 1055: LOE 2b, GR B). Both extracts are contained in high-quality medicinal products, which were originally registered in Germany, but in the meantime have been registered internationally. For several products without marketing authorization and therefore without quality approval by regulatory authorities, quality deficiencies may be the reason for the deviating results regarding efficacy. Short descriptions of product quality cannot replace extensive product specifications of drug registration dossiers. The quality of a herbal medicinal product depends on optimally concerted procedures applied from herbal substance to the final product. Optimization of procedures and thereby product quality clearly benefits from decades of technical expertise. For example, the oldest herbal medicinal *Cimicifuga racemosa* product (marketed since 1956) is manufactured according to standardized procedures, which are part of the descriptions in drug registration dossiers. These are approved by regulatory authorities in a multitude of different countries, but not accessible to the public. Available data on this long-standing product is consistently positive.

It is important to differentiate between clinical trials with standardized registered herbal remedies whose pharmaceutical quality has been checked by regulatory authorities and clinical trials with other products.

## 5. Conclusion

Ideally, therapeutic decisions should be based on consistent scientific evidence. Consistently positive data regarding efficacy and safety have clearly been demonstrated for the isopropanolic *Cimicifuga racemosa* extract (iCR) and the ethanolic extract (BNO 1055) in the treatment of natural climacteric complaints. A multitude of clinical studies including more than 11,000 patients has been identified for the iCR extract, which demonstrates not only a good safety profile but also consistent confirmatory evidence for efficacy. Several clinical trials including more than 500 patients were identified for the BNO 1055 extract, demonstrating a good safety profile and consistent exploratory evidence for efficacy. Both extracts are in compliance with the recent *Cimicifuga racemosa* monograph of the Herbal Medicinal Products Committee and the corresponding preparations are registered as herbal medicinal products in many countries in and outside Europe.

This is the first review of a herbal treatment option based on a differentiation by extract and indication and therefore meets the distinctive characteristics of phytotherapy. The evidence concerning efficacy is favorable and consistent only for those *Cimicifuga racemosa* products which hold a marketing authorization for the treatment of climacteric complaints.

## Figures and Tables

**Table 1:** Inclusion, exclusion, and stratification criteria for the identification of the literature for the evaluation of the efficacy and safety of *Cimicifuga racemosa*.

	Efficacy	General safety	Safety in estrogen-sensitive organs	Liver safety
*Inclusion criteria *				
(1) Medical application of Black Cohosh	+	+	+	+
(2) 3-month treatment period at a minimum	+			
(3) Full publication of clinical studies	+	+	+	+
(4) Efficacy in natural climacteric complaints (neurovegetative, psychic)	+			
(5) Occurrence and frequency of adverse events		+	+	+
(6) Influence on hormone parameters, breast, and uterus			+	
(7) Influence on liver function parameters				+

*Exclusion criteria *				
(1) Preparations containing more than two ingredients	+	+	+	+
(2) Medication-induced complaints (e.g., tamoxifen)	+			
(3) Use outside the registered indication (bone metabolism, anxiety disorder, and cognition)	+	+	+	+
(4) Prevention, including tertiary prevention of sequelae	+			
(5) Publications in languages other than English/German	+	+	+	+

*Stratification criteria *				
(1) Sorting by type of extract	+	+	+	+
(2) Differentiation between registered medicinal products versus other products	+	+	+	+

**Table 2:** Clinical studies between 2000 and 2012 on the efficacy of *Cimicifuga racemosa* in neurovegetative and psychic climacteric complaints, differentiated by extract and regulatory status (registered medicinal product-not registered as medicinal product).

Extract DER; extractant/standardization; brand name; manufacturer	Regulatory status	First author, year of publication	Design	Number of patients	Dose (*Cimicifuga*-drug/day or control/day); duration of application	Main results	Impact on evidence
Isopropanolic special extract (iCR) DER 6–11 : 1; 40% Isopropanol Remifemin; Schaper & Brümmer, Germany	Medicinal product (EU and outside EU)	Liske, 2002	RCT	76: standard dose 76: high dose	39 mg 127 mg 3 months (*n* = 152) up to 6 months (*n* = 149)	Significant improvement of the KMI; responder rate (KMI < 15) after 3 months approx. 70%; high dose during menopausal transition significantly superior; no influence on hormones and vaginal cytology	*⊕*
		Osmers, 2005	RCT	153: iCR 151: placebo	40 mg 3 months	Significant improvement of the MRS-total score versus placebo; strongest effect in vasomotor complaints (hot flushes, sweatings, and sleep disorders) and higher superiority during menopausal transition	*⊕⊕*
		Nappi, 2005	RCT	32: iCR 32: HT (TTSE_2_)	40 mg 25 *μ*g + Progesterone 3 months	In both groups comparably strong, significant improvement of vasomotor complaints, anxiety, and depression; no influence of iCR on hormones, liver function, and endometrium	*⊕*
		Bai, 2007	RCT	122: iCR 122: tibolone	40 mg 2.5 mg 3 months	Comparably strong, significant reduction of the KMI in both groups; with better safety of iCR: superiority of iCR in the benefit-risk ratio	*⊕⊕*
		Schmidt, 2005	ONC	502	40 mg 3 months	Significant improvement of the modif. KMI, most pronounced reduction of hot flushes and sweatings	*⊕*
		Vermes, 2005	ONC	2,016	40 mg 3 months	Significant improvement of the KMI, strongest for hot flushes, sweatings, sleeping difficulties, and anxiety	*⊕*
		Julia Molla, 2009	ONC	122	40 mg 3 months	Significant increase in quality of life (Cervantes-HR-QoL-Scale), especially in the domains menopause/health and psyche	*⊕*

Isopropanolic special extract (iCR) (see previously) with *Hypericum perforatum* extract; standardized to 1 mg triterpene glycosides and 0.25 mg total hypericin Remifemin plus; Schaper & Brümmer, Germany	Medicinal product (EU)	Uebelhack, 2006	RCT	151: iCR + Hyp 150: placebo	120 mg (week 1–8) 60 mg (week 9–16) 4 months	Significant superiority versus placebo in the improvement of MRS- and HAMD-Scores	*⊕⊕*
		Briese, 2007	OC	3,114: iCR + Hyp 3,027: iCR	60–120 mg 40 mg 6 months (*n* = 6,141) up to 12 months (*n* = 736)	In both groups, significant improvement of the MRS-total score and -subscore *Psyche*; for psychic complaints iCR + Hyp is significantly more effective than iCR monotherapy	*⊕⊕*

No data on extractant and DER *Cimicifuga*-extract with *hypericum* extract, standardized to 1 mg terpene glycosides and 0.25 mg hypericin Gyno-plus; Jin-Yang Pharm, Korea	Medicinal product (Korea)	Chung, 2007	RCT	47: CR + Hyp 42: placebo	According to SPC 3 months	Significant superiority in the improvement of the KMI versus placebo; no influence on hormones and vaginal cytology; significant increase of HDL in the CR + Hyp-group	*⊕⊕*

Ethanolic extract (CR BNO 1055) DER 5–10 : 1; 58% ethanol Klimadynon; Bionorica, Germany	Medicinal product (EU and outside EU)	Wuttke, 2003	RCT	20: CR 22: HT (CE) 20: placebo	40 mg 0.6 mg 3 months	CR: comparable improvement of the MRS score to CE and superiority versus placebo, however, not significant; CR without effects on the endometrium, positive influence on bone markers and vaginal cytology	*⊕*
		Wuttke, 2006	RCT-Reana-lysis	See Wuttke, 2003	See Wuttke, 2003	Exploratory reanalysis: significant superiority of CR versus placebo in regard to improvement of daily sweats, nightly waking, and MRS subscores *major climacteric complaints, somatic complaints*, and *mental score *	*⊕*
		Rauš, 2006	ONC	400	40 mg 12 months	No influence on the endometrium; significant improvement of MRS II and MRS II 4-week weighted score of hot flushes	*⊕*

Ethanolic extract (Cr 99) 4.5–8.5 : 1; 60% Ethanol *Cimicifuga* Generic, Klimadynon Uno; Kolkmann, Bionorica, Germany	Medicinal product (EU)	Frei-Kleiner, 2005	RCT	83: CR 44: placebo	42 mg 3 months	Superiority versus placebo in KMI and weekly weighted score of hot flushes, which is significant in patients with baseline KMI > 20	*⊕*

Ethanolic extract No information on DER, 50% ethanol Remixin, Mikro-Gen, Turkey	Medicinal product (Turkey)	Oktem, 2007	RCT	60: CR 60: Fluoxetine	40 mg 20 mg 6 months	Significant improvement in modified KMI, Beck's Depression Scale, and RAND-36 QoL in both groups; CR: significant superiority versus Fluoxetine in KMI and monthly scores for hot flushes and night sweats	*⊕*

Ethanolic extract (Ze 450) DER 4.5–8.5 : 1; 60% ethanol Cimifemin; Zeller, Switzerland	Medicinal product (Switzerland)	Kaiser, 2008	RCT	60: standard dose 60: high dose 60: placebo	40 mg 80 mg 3 months	CR: significant superiority versus placebo in KMI high dose: significant superiority versus standard dose in KMI	*⊕*

Ethanolic extract No information on DER, 70% ethanol, 2.5% triterpene glycosides Pure World, USA	No medicinal product (USA)	Newton, 2006	RCT	80: CR 76: Multibotanical 79: Multibotanical + Soy 32: CEE + MPA 84: placebo	160 mg extract? 200 mg + 9 further ingredients 0.625 mg + 2.5 mg 12 months	No significant differences between CR and placebo for vasomotor symptoms per day, symptom intensity, Wiklund Vasomotor Symptom Subscale Score	*⊖*

Ethanolic extract 20 : 1; 75% ethanol, standardized to 3.64 mg triterpene glycosides University of Illinois/National Institutes of Health, USA	No medicinal product, only study medication (USA)	Geller, 2009	RCT	22: CR 22: red clover 23: CEE/MPA 22: placebo	128 mg 120/378 mg 0.625/2.5 mg 12 months	No significant improvement of the frequency of vasomotor complaints by CR versus placebo	*⊖*

No information on extractant and DER CimiPure 2.5%, Pure World, Inc., USA	No medicinal product, food supplement (USA)	Ruhlen, 2007	ONC	61	80 mg extract? 3 months plus 3 months wash out	Significant improvement of the KMI by CR, increase after wash out; no effects in serum estrogen markers, pS2, or cellular morphology of NAF	*⊕*

CE: conjugated estrogens; CEE: conjugated equine estrogens; CR: *Cimicifuga racemosa*; DER: drug-extract ratio; HAMD: Hamilton Rating Scale for Depression; HR-QoL: Health-Related Quality of Life; HT: hormone therapy; Hyp: *Hypericum perforatum*; iCR: isopropanolic *Cimicifuga racemosa* special extract; KMI: Kupperman Menopause Index; MPA: medroxy progesterone acetate; MRS: Menopause Rating Scale; NAF: nipple aspirate fluid; OC: open controlled; ONC: open noncontrolled; RAND-36 QoL: RAND-36 measure of Health-Related Quality of Life; pS2: pS2 gene; RCT: randomised controlled trial; SPC: summary of product characteristics; TTSE_2_: transdermal estradiol.

Impact on Evidence for efficacy: *⊕⊕*: confirmatory evidence; *⊕*: exploratory evidence; *⊖*: no evidence.

**Table:** (a)

All studies	Registered herbal medicinal product	
	Yes	No
Evidence for efficacy		
Yes	15	1
No	0	2

*n* = number of studies, *P* = 0.0008 (Chi^2^-test).

**Table:** (b)

Subset of confirmatory studies	Registered herbal medicinal product	
	Yes	No
Evidence for efficacy		
Yes	5	0
No	0	2

*n* = number of studies, *P* = 0.008 (Chi^2^-test).

Registered = holding a marketing authorization.

**Table 4:** Clinical studies between 2000 and 2012 on the safety of *Cimicifuga racemosa*, differentiated by extract and regulatory status (registered medicinal product-not registered as medicinal product).

Extract DER; extractant/standardization; brand name; manufacturer	Regulatory status	First author, year of publication	Design	Number of patients	Dose (*Cimicifuga*-drug/day or control/day); duration of application	General safety	Safety on estrogen sensitive organs	Liver safety
Isopropanolic special extract (iCR) DER 6–11 : 1; 40% isopropanol Remifemin; Schaper & Brümmer, Germany	Medicinal product (within and outside EU)	Naser, 2011	M-A (from 5 RCTs)	1,117: iCR or iCR + Hyp	40–128 mg 3–6 months			*↔*: AST, ALT, and GGT no evidence for hepatotoxicity
		Jacobson, 2001	RCT	Breast cancer patients (partly with Tam) 42: iCR 43: placebo	40 mg 60 days	Few serious AEs only in Tam patients	*↔*: FSH, LH	
		Liske, 2002	RCT	76: standard dose 76: high dose	39 mg 127 mg 3 months (*n* = 152) to 6 months (*n* = 149)	Good/very good tolerability in both groups (82–100%), no serious AEs, *↔*: routine biochemistry and haematology	*↔*: E_2_, LH, FSH, PRL, SHBG, and vaginal cytology	
		Osmers, 2005	RCT	153: iCR 151: placebo	40 mg 3 months	AEs: no significant differences, no serious AEs		*↔*: AST, ALT, and GGT
		Nappi, 2005	RCT	32: iCR 32: HT (TTSE_2_)	40 mg 25 *μ*g + progesterone 3 months	iCR: *↔*: CORT, CHOL, TGL ↑: HDL ↓: LDL	iCR: *↔*: E_2_, FSH, LH, PRL, and endometrial thickness; no vaginal bleeding	*↔*: AST, ALT
		Bai, 2007	RCT	122: iCR 122: Tibolone	40 mg 2.5 mg 3 months	iCR: significantly fewer AEs, no serious AEs, *↔*: BW, haematology, blood chemistry, urinalysis, AP, and CRP	iCR: no postmenopausal bleeding; *↔*: endometrial thickness	*↔*: AST, ALT, and GGT
		García-Pérez, 2009	OC	45: iCR 37: untreated	40 mg 3 months	Good tolerability, *↔*: routine biochemistry, lipids, PTH ↓: NTx ↑: AP	*↔*: E_2_, FSH, LH, and TEST	

Isopropanolic special extract (iCR) DER 6–11 : 1; 40% isopropanol Remifemin; Schaper & Brümmer, Germany	Medicinal product (within and outside EU)	Pockaj, 2004	ONC	21 (13 with breast cancer)	40 mg 4 weeks	1 AE (joint pain)	*↔* transcriptional-activation assay S. cerevisiae PL3	
		Schmidt, 2005	ONC	502	40 mg 3 months	Tolerability very good, no AEs		
		Vermes, 2005	ONC	2,016	40 mg 3 months	12.1% AEs, specified by *n* = 35; mostly stiffening of extremities, gastric pain, and allergic reactions		
		Lindén-Hirschberg, 2007	ONC	74	40 mg 6 months	No serious AEs *↔*: CHOL, TGL, and IGF-I	*↔*: mammographic breast density (visual assessment), breast cell proliferation, endometrial thickness, and SHBG	
		Reame, 2008	ONC	6	40 mg 3 months		*↔*: spontaneous LH pulsatility	
		Rostock, 2011	ONC	50 with breast cancer and Tam	20–80 mg 6 months	Tolerability good/very good: 90% AEs not linked to iCR but Tam		
		Lundström, 2011	ONC/RCT Reana-lysis	64: iCR 43: E_2_/NETA 49: tibolone 53: placebo	40 mg 2 mg/1 mg 2.5 mg 6 months		For iCR: *↔*: mammographic breast density (digitized assessment)	
		Rebbeck, 2007	C-C iCR	1,524 controls: 76: CR or iCR 949 breast cancer cases: 25: CR or iCR	No data		iCR/CR: ↓: risk for breast cancer	

Isopropanolic special extract (iCR) DER 6–11 : 1; 40% isopropanol Remifemin; Schaper & Brümmer, Germany	Medicinal product (within and outside EU)	Obi, 2009	C-C iCR	6,646 controls: 320: iCR 89: other CR 3,257 breast cancer cases: 112: iCR 34: other CR	No data		iCR: ↓: risk for breast cancer other CR: *↔* risk for breast cancer	
		Henneicke-von Zepelin, 2007	COH	18,861 breast cancer cases 1,102: iCR	No data mean follow-up 4.6 years		iCR: ↓: risk for breast cancer recurrences	

Isopropanolic special extract (iCR) (see previously) with *Hypericum perforatum* extract; standardized to 1 mg triterpene glycosides and 0.25 mg total hypericin Remifemin plus; Schaper & Brümmer, Germany	Medicinal product (EU)	Uebelhack, 2006	RCT	151: iCR + Hyp 150: placebo	120 mg (Week 1–8) 60 mg (Week 9–16) 4 months	AEs: no significant differences, no serious AEs, *↔*: haematology, biochemical and hormonal parameters		
		Briese, 2007	OC	3,114: iCR + Hyp 3,027: iCR	60–120 mg 40 mg 6 months (*n* = 6,141) up to 12 months (*n* = 736)	0.16% possibly treatment related AEs, no serious AEs, excellent/good tolerability >90–98%		

No data on extractant and DER *Cimicifuga*-extract with *hypericum*-extract, standardized to 1 mg terpene glycosides and 0.25 mg hypericin Gyno-plus; Jin-Yang Pharm, Korea	Medicinal product (Korea)	Chung, 2007	RCT	47: CR + Hyp 42: placebo	As per SPC 3 months	CR: *↔*: routine chemistry, CHOL, LDL, and TGL ↑: HDL AEs: 8 patients (versus 6) with 3 (versus 2) drop outs	CR: *↔*: E_2_, FSH, LH, VMI no uterine bleeding in patients with gynecological disorders	

Ethanolic extract (CR BNO 1055) DER 5–10 : 1; 58% ethanol Klimadynon; Bionorica, Germany	Medicinal product (EU and outside EU)	Wuttke, 2003	RCT	20: CR 22: HT (CE) 20: placebo	40 mg 0.6 mg 3 months	CR: AEs: no significant differences, no serious AEs ↑: TGL, AP ↓: CrossLaps	CR: *↔*: E_2_, FSH, LH, PROG, and endometrial thickness ↑: VMI	
		Wuttke, 2006	RCT Reana-lysis	See Wuttke, 2003	See Wuttke, 2003	CR: nonserious AEs in 6 patients		
		Wuttke, 2006	RCT Reana-lysis	See Wuttke, 2003	See Wuttke, 2003	See Wuttke 2003; CR: ↑: CHOL, HDL, and LDL *↔*: HR, BP, BW, CREA, urea, uric acid, PROT, Na, K, Ca, Fe, BILI, GLU, blood count, PTT, and INR	See Wuttke 2003; CR: ↓: SHBG	CR: *↔*: AST, ALT, and GGT
		Hernández-Muñoz, 2003	OC	Breast cancer patients 90: Tam + CR 46: Tam	20 mg + 40 mg 20 mg 12 months	CR + Tam: 4 AEs; Tam: 7 AEs; no serious AEs		
		Rauš, 2006	ONC	400	40 mg 12 months	*↔*: HR, BP, BW, CHOL, HDL, LDL, TGL, INR, GLU, uric acid, BILI, and GLU	*↔*: endometrial biopsy, endometrial thickness, mammographic breast density, E_2_, FSH, and LH	*↔*: AST, ALT, and GGT
		Nasr, 2009	ONC	87	40 mg 12 months	*↔*: BP, BW, BILI, ALB, AP, PT, and PTC		*↔*: hepatic blood flow, AST, ALT, and GGT

Ethanolic extract (Cr 99) 4.5–8.5 : 1; 60% Ethanol *Cimicifuga*-Generic, Klimadyon Uno; Kolkmann, Germany	Medicinal product (EU)	Frei-Kleiner, 2005	RCT	83: CR 44: placebo	42 mg 3 months	AEs: no significant differences; For CR: *↔*: HB, AP, and CREA	*↔*: vaginal karyopyknotic index	*↔*: AST, ALT

Ethanolic extract No information on DER, 50% ethanol Remixin, Mikro-Gen, Turkey	Medicinal product (Turkey)	Oktem, 2007	RCT	60: CR 60: Fluoxetine	40 mg 20 mg 6 months	CR: significantly fewer AEs versus fluoxetine		

Ethanolic extract (Ze 450) DER 4.5–8.5 : 1; 60% ethanol Cimifemin; Zeller, Switzerland	Medicinal product (Switzerland)	Kaiser, 2008	RCT	60: standard dose 60: high dose 60: placebo	40 mg 80 mg 3 months	CR: 8 mild AEs, without relation to dose; placebo: 6 mild AEs		

Ethanolic extract No information on DER, 70% ethanol, 2.5% triterpene glycosides Pure World, USA	No medicinal product (USA)	Newton, 2006	RCT	80: CR 76: Multibotanical 79: Multibotanical + Soy 32: CEE + MPA 84: placebo	160 mg extract? 200 mg + 9 further ingredients 0.625 mg + 2.5 mg 12 months	CR: significantly fewer menstrual complaints/breast discomfort versus CEE + MPA, no significant differences regarding other AEs in all groups		
		Spangler, 2007	RCT Reana-lysis	See Newton, 2006	See Newton, 2006	CR: *↔*: CHOL, HDL, LDL, TGL, INS, GLU, and FIBR		
		Reed, 2008	RCT Reana-lysis	See Newton, 2006	See Newton, 2006		CR: *↔*:, E_2_, FSH, LH, SHBG, bleeding patterns, and VMI	

Ethanolic extract 20 : 1; 75% ethanol, 64 mg/capsule standardized to 3.64 mg triterpene glycosides University of Illinois/National Institutes of Health, USA	No medicinal product, pure study medication (USA)	Geller, 2009	RCT	22: CR 22: red clover 23: CEE/MPA 22: placebo	128 mg 120/378 mg 0.625/2.5 mg 12 months	CR: AEs no significant differences, no serious AEs *↔*: CHOL, HDL, LDL, DXA, PT, TSH, AP, blood count, urinalysis, and serum chemistry	CR: *↔*: E_1_, E_2_, FSH, LH, SHBG, TEST, TVU, PAP, endometrial biopsy, and mammogram	CR: *↔*: AST, ALT

No information on extractant and DER, 32 mg/capsule standardized to 5.6% triterpene glycosides University of Illinois/National Institutes of Health, USA	No medicinal product, pure study medication (USA)	Amsterdam, 2009	RCT	15: CR 13: placebo	32–64 mg 12 weeks	AEs: no significant differences		

Ethanolic extract No information on DER, 70% ethanol Pure World, USA	No medicinal product (USA)	Johnson, 2003	OC	7	32/64/128 mg extract single application			No mercapturate conjugates found in urine

No information on extractant and DER CR-extract 1,090 mg, standardized to 0.9% triterpene glycosides Solaray, USA	No medicinal product (USA)	Gurley, 2005	OC	12 (6 women)	2180 mg extract 28 days	*↔*: CYP3A4/5, CYP 1A2, CYP2E1, and CYP2D6		

No information on extractant and DER CR-extract 20 mg, standardized to 2.5% triterpene glycosides Enzymatic Therapy, USA	No medicinal product (USA)	Gurley, 2006	OC	16 (8 women)	40 mg extract? 14 days	*↔*: p-gp		

No information on extractant and DER CimiPure 2.5% Pure World, Inc., USA	No medicinal product, food supplement (USA)	Ruhlen, 2007	ONC	61	80 mg extract? 3 months plus 3 months wash out		*↔*: E_2_, FSH, LH, pS2, and cellular morphology in NAF	

No information on extractant and DER, standardized to 2.5% actein no data, Italy	No data (Italy)	Firenzuoli, 2011	ONC	107	500–1,000 mg extract? min. 12 months	Minor transient AEs in 9 patients *↔*: LEU, BILI, AP, ALB, and INR		*↔*: AST, ALT, and GGT

Ethanolic extract, No information on DER, 75% ethanol, 32 mg extract/capsule, standardized to 7% triterpene glycosides Pure World/Naturex, USA	No medicinal product(USA)	Van Breemen, 2010	ONC	15	32/64/128 mg extract Single application	*↔*: GLU, AP, BILI, and TSH	*↔*: E_1_, E_2_, FSH, LH, SHBG, and TEST	*↔*: AST, ALT, and GGT

Different CR preparations USA	No data (USA)	Brasky, 2010	C-C	880 breast cancer patients: 21: CR 34,136 noncases: 964: CR	No data		CR not associated with breast cancer risk	

AE: adverse effect; ALB: serum albumin; ALT: alanine aminotransferase; AP: alkaline phosphatase; AST: aspartate aminotransferase; BILI: total bilirubin; BP: blood pressure; BW: body weight; Ca: Calcium; C-C: case-control study; CE: conjugated estrogens; CEE: conjugated equine estrogens; CHOL: total cholesterol; COH: cohort study; CORT: cortisol; CR: *Cimicifuga racemosa*; CREA: creatinine; CrossLaps: C-terminal telopeptides of collagen type I; CRP: C-reactive protein; CYP: cytochrome P450; DER: drug-extract ratio; DXA: dual-energy X-ray absorptiometry; E_1_: estrone; EU: European Union; E_2_: 17*β*-estradiol; Fe: iron; FIBR: fibrinogen; FSH: follicle-stimulating hormone; GGT: *γ*-glutamyl transpeptidase; GLU: glucose; HB: hemoglobin; HDL: high-density lipoprotein cholesterol; HR: heart rate; HT: hormone therapy; Hyp: *Hypericum perforatum*; iCR: isopropanolic *Cimicifuga racemosa* special extract; IGF-I: insulin-like growth factor I; INR: international normalized ratio; INS: insulin; K: potassium; LDL: low-density lipoprotein cholesterol; LEU: total leukocyte count; LH: luteinizing hormone; M-A: meta-analysis; MPA: medroxyprogesterone acetate; Na: sodium; NAF: nipple aspirate fluid; NTx: N-telopeptide of type I collagen; OC: open controlled; ONC: open noncontrolled; PAP: Pap smear; p-gp: p-glycoprotein; PRL: prolactin; PROG: progesterone; PROT: total protein; pS2: pS2 gene; PT: prothrombin time; PTC: prothrombin concentration; PTH: parathyroid hormone; PTT: activated thromboplastin time; RCT: randomised controlled trial; SHBG: sex hormone-binding globulin; SPC: summary of product characteristics; Tam: tamoxifen; TEST: testosterone; TGL: triglycerides; TSH: thyroid-stimulating hormone; TTSE_2_: transdermal estradiol; TVU: transvaginal ultrasound; VMI: vaginal maturation index.

*↔*: No clinically relevant changes; ↑: increase; ↓: decrease.
